# A Simple Procedure for Mesh Suture Fixation in Robotic Inguinal Hernia Repair: A Technical Report

**DOI:** 10.7759/cureus.81982

**Published:** 2025-04-09

**Authors:** Ippei Yamana, Takahisa Fujikawa, Keiji Nagata, Kei Harada, Suguru Hasegawa

**Affiliations:** 1 Surgery, Kokura Memorial Hospital, Kitakyushu, JPN; 2 Gastroenterological Surgery, Fukuoka University Hospital, Fukuoka, JPN

**Keywords:** inguinal hernia surgery, laparoscopic inguinal hernia, mesh fixation, robotic inguinal hernia repair, robotic surgical procedure

## Abstract

Robotic surgery has become increasingly popular worldwide; however, robotic inguinal hernia repair remains less common outside the United States. As a result, the standardization of robotic transabdominal preperitoneal repair (R-TAPP) techniques has not yet been established and requires further validation. In traditional laparoscopic transabdominal peritoneal repair, mesh fixation is typically achieved using a tucker. In contrast, R-TAPP necessitates the use of sutures for mesh fixation, which can be challenging due to the risk of peritoneal drooping - especially in cases involving the abandonment of the hernia sac technique. This report presents a straightforward technique for mesh suture fixation in R-TAPP that enhances the efficiency of the procedure by streamlining the fixation process on the ventral side of the mesh. In conclusion, this method significantly improves the overall efficiency of R-TAPP, making it a valuable addition to surgical practice.

## Introduction

Recently, robotic surgery has become increasingly common in gastrointestinal surgery. Compared to traditional laparoscopic surgery, robotic surgery offers benefits such as reduced hand movements and a three-dimensional, magnified view. Robotic transabdominal preperitoneal repair (R-TAPP) is also widely performed in Europe and the USA, with many reports highlighting its effectiveness [1-5].

In Europe and the United States, the standard method for creating a peritoneal flap during laparoscopic and robotic procedures typically involves making a ventral peritoneal incision [1-5]. In contrast, the abandonment of the hernia sac technique, which does not require ligation of the hernia sac for indirect hernias, has been frequently employed in Japan [6-9].

In laparoscopic transabdominal peritoneal repair (L-TAPP), mesh fixation has been achieved using a tucker, whereas in R-TAPP, mesh fixation is performed with sutures. Initially, peritoneal drooping posed challenges for mesh suture fixation, making the process difficult and time-consuming - particularly in cases involving abandonment of the hernia sac technique. Therefore, we present a simple method for mesh suture fixation in R-TAPP.

## Technical report

Introduction for R-TAPP

At our institution, we have introduced and evaluated R-TAPP for inguinal hernias since July 2023. Between October 2023 and October 2024, we performed R-TAPP in 16 cases using this technique. All operations were performed by two board-certified surgeons who completed the L-TAPP before performing the R-TAPP under a protocol designed at our hospital. With regard to R-TAPP, one of them performed 10 cases, while the other performed six. This study was approved by the Institutional Review Board of our institution.

Surgical technique

**Figure 1:**
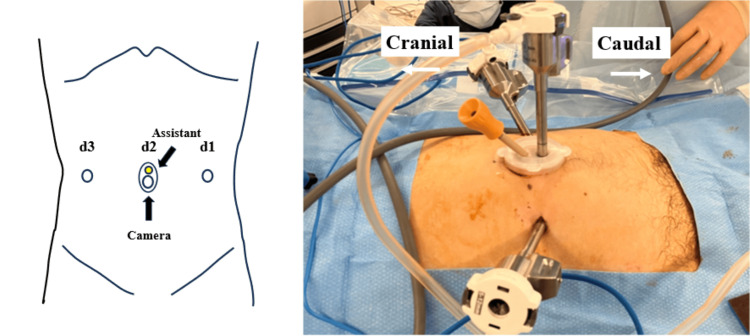
Trocar placement An 8-mm camera trocar and a 5-mm assistant trocar were inserted into the lap protector, with two 8-mm working trocars positioned at least 8 cm away from the umbilical site. Left-side image credit: Ippei Yamana d1: 8-mm trocar; d2: 0504 LAP PROTECTOR mini-mini and EZ Access (Hakko, Tokyo, Japan) (8-mm trocar + 5-mm assistant trocar); d3: 8-mm trocar

All procedures were performed using the Da Vinci Xi surgical system (Intuitive Surgical, Inc., Sunnyvale, CA, USA). Patients were positioned in the Trendelenburg position at an angle of 13 degrees. A 2-cm umbilical skin incision was made, and a 0504 LAP PROTECTOR mini-mini oval type and EZ Access (Hakko, Tokyo, Japan) were placed at the umbilical site. An 8-mm camera trocar and a 5-mm assistant trocar were inserted into the lap protector, with two 8-mm working trocars positioned at least 8 cm away from the umbilical site (Figure 1). 

Fenestrated forceps were used with the left hand, while Maryland bipolar forceps were employed with the right hand. The Maryland bipolar forceps were connected to a VIO 300D electrosurgical generator (Erbe USA, Marietta, GA, USA), set to cutting mode.

**Figure 2:**
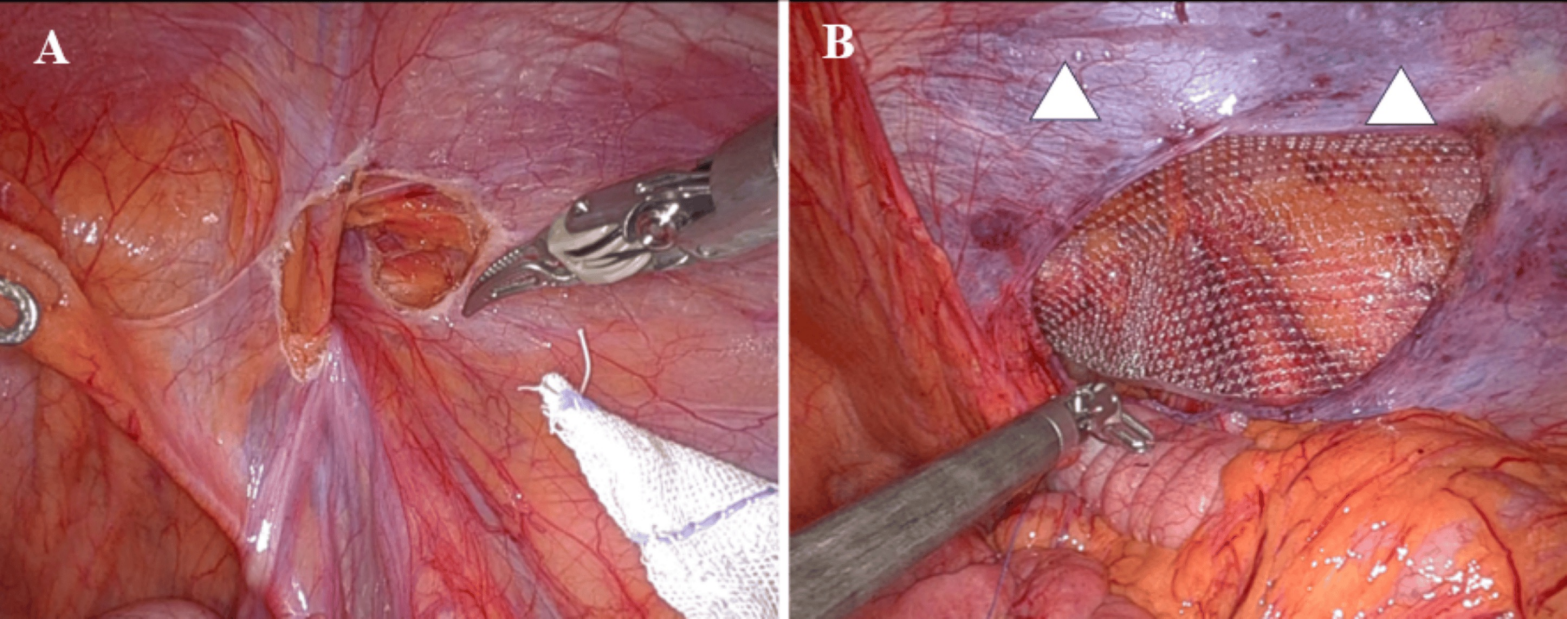
Abandonment of hernia sac technique (A) For indirect hernias, we performed the abandonment of the hernia sac technique, ensuring that the upper margin of the peritoneal incision was aligned with the upper margin of the hernia sac. (B) The drooping of the peritoneum obscures the area for mesh suture fixation, making the suturing process more difficult (arrowheads: mesh suture fixation site).

**Figure 3:**
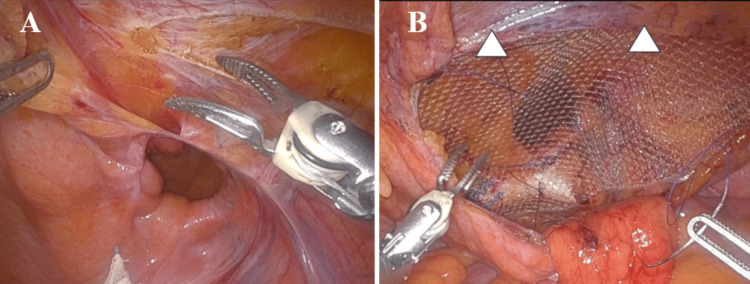
Ventral peritoneal incision (A) For direct hernias, a ventral peritoneal incision was made, with its upper margin positioned 3 cm above the upper margin of the hernia sac. (B) In cases of ventral peritoneal incision, the absence of peritoneal drooping facilitates easier mesh suture fixation (arrowheads: mesh suture fixation site).

For indirect hernias with a deep hernia sac, we performed the abandonment of the hernia sac technique, ensuring that the upper margin of the peritoneal incision was aligned with the upper margin of the hernia sac (Figures 2A-2B). For direct hernias or indirect hernias with a shallow hernia sac, a ventral peritoneal incision was created, with its upper margin positioned 3 cm above the upper margin of the hernia sac (Figures 3A-3B).

**Figure 4:**
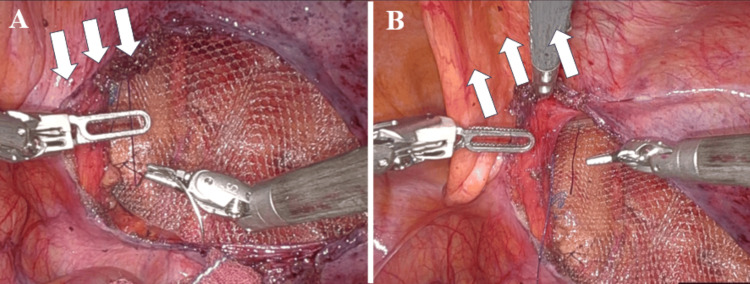
Elevation of drooped peritoneum (A) During the suture fixation of the ventral side of the mesh, particularly after performing an abandonment of the hernia sac technique, it was challenging to secure the sutures due to the drooping peritoneum (arrows: peritoneum drooping). (B) To assist the operator, the assistant elevated the drooped peritoneum using forceps through the 5-mm assistant trocar located in the umbilical region (arrows: peritoneum elevation).

We used a 3DMax™ Light Mesh (Becton Dickinson, Warwick, RI, USA), which was sutured and secured to the left and right sides of the inferior epigastric vein and Cooper’s ligament. During the suture fixation of the ventral side of the mesh, particularly after performing the abandonment of the hernia sac technique, it was challenging to secure the sutures due to the drooping peritoneum. To assist the operator, the assistant elevated the drooping peritoneum using forceps through the 5-mm assistant port located in the umbilical region (Figures 4A-4B; Video 1).

**Video 1:** Simple procedure of mesh suture fixation for robotic inguinal hernia repair During suture fixation of the ventral mesh after the abandonment of the hernia sac technique, securing the sutures was difficult due to the drooping peritoneum. To assist, the assistant elevated the peritoneum with forceps through the 5-mm assistant port in the umbilical region.

## Discussion

Robotic surgery has gained considerable popularity worldwide [10]. However, robotic inguinal hernia repair remains less common outside the United States [11,12]. In Japan, inguinal hernia repair is predominantly performed using either an anterior approach [13] or a laparoscopic approach [14], with R-TAPP not yet being covered by insurance. As a result, the standardization of R-TAPP techniques has not been established and requires further verification.

In Europe and the United States, the prevailing method for creating a peritoneal flap during laparoscopic and robotic procedures typically involves a ventral peritoneal incision [1-5]. This technique is well-established and widely practiced. In contrast, Japan has developed an abandonment of the hernia sac technique, which has gained popularity among many surgeons [6-9]. The ventral peritoneal incision is advantageous, as it allows for straightforward and efficient inversion of the hernia sac in cases of direct hernias. However, this process is more complicated in indirect hernias with deep hernia sacs, and successful inversion is difficult. If inversion cannot be achieved, the surgeon must resort to ligating or dissecting the hernia sac, complicating the surgical procedure.

The abandonment of the hernia sac technique utilized in Japan offers distinct advantages, as it does not require ligation of the hernia sac. This method is also reproducible, involving a systematic approach in which the sac is first separated in an annular manner. While the ventral peritoneal incision rarely results in peritoneal drooping, the abandonment of the hernia sac technique can lead to a lower incision on the ventral side, potentially causing the peritoneum to droop and obstruct the surgical field. Therefore, it is essential to adequately elevate the peritoneum to facilitate effective mesh fixation.

To date, there have been no reports on how to deal with perineal droop in R-TAPP. To resolve the drooping peritoneum during R-TAPP, we implemented a straightforward method using a lap protector at the umbilical region. The umbilical skin incision is 2 cm, almost the same size as the usual camera port skin incision. An assistant elevates the peritoneum with forceps during mesh suturing, facilitating secure fixation and reducing overall time. Based on our experience, before this method, we had sutured the drooped peritoneum to the abdominal wall on the ventral side and then sutured the mesh, which required a great deal of time. However, the technique enables a shorter fixation time compared to those without the lap protector (mean 7.1 minutes vs. mean 21.9 minutes). By strategically positioning the assistant port on the caudal side of the camera port, we minimize interference with the robotic arm. This positioning allows for easy and effective ventral elevation of the peritoneum. In addition, it can be used not only to elevate the drooping peritoneum in a ventral peritoneal incision, but also for thread insertion and removal, thread cutting, and peritoneal traction during peritoneal suture closure.

R-TAPP is not currently covered by insurance in Japan, leading to a lack of standardized surgical techniques and few cases. However, once insurance coverage is established, the effective abandonment of the hernia sac technique is expected to be increasingly used for indirect hernias. In such scenarios, the implementation of this procedure is expected to be safe and significantly reduce operative time, thereby improving patient outcomes.

The study's retrospective cohort design has inherent biases, and its findings are limited by the practices of two surgeons and a small sample size. Larger studies involving multiple surgeons are needed to validate the benefits of this technique. Additional data and clinical experience will be essential to further validate the benefits of this technique.

## Conclusions

We introduce an effective method for mesh suture fixation in R-TAPP. Elevating the peritoneum via the assistant's port allows for more efficient and accurate suture fixation of the ventral portion of the mesh. This method simplifies the suture fixation process, significantly improves the flow of the procedure, and is expected to gain high versatility in the future due to its simplicity and reproducibility. Consequently, this technique enhances the overall efficiency and effectiveness of R-TAPP, potentially improving surgical outcomes and reducing operative duration.
